# Vitis Vinifera Radix, as a Diuretic

**Published:** 1856-08

**Authors:** A. J. Simmons

**Affiliations:** Bankston, Monroe Co., Ga.


### ARTICLE IV.

BANKSTON, Monroe Co., Ga.,
May 8th, 1856.

### *Vitis Vinifera Radix, as a Diuretic.*

GENTLEMEN—From a considerable experience, I have found the Grape Vine Root to be one of the best diuretics known to me. Scarlet fever has been in my section for some length of time, having many cases of genuine *Anasarca* to treat as a sequel of that disease, I have given the Grape Vine Root a fair trial. In a number of cases nothing else prescribed, the water moving off rapidly.

I have the root procured, and then placed upon a heated oven-lid and there burnt into ashes.

℞. Two table spoonsful of the ashes, pour on a pint of boiling water. The patient drinks it *ad libitum.*

Another prescription often used—℞. Two table spoonsful of the ashes, ℥ii bitartrate potass., pour on a pint of boiling water. Taken *ad libitum.*

CASE.—A case of *Anasarca* of the lower extremities—the subject was a stout negro woman, *enciente* some months, plethoric, robust, hearty woman. Her legs, thighs and left labia much enlarged. ℞ Grape vine root, two table spoonsful, bitartrate potass., ℥ii. Pour on a pint of boiling water—patient taken the above prescription, *ad libitum*, consuming about a pint a day, in less than five days the swelling had completely subsided.

I mention this case for the benefit of the young practitioner as he is often at a loss how to treat such cases.

Very Respectfully,

A. J. SIMMONS, M. D.

